# Complete and long-lasting response to immunotherapy in a stage IV non-small cell lung cancer with brain metastasis

**DOI:** 10.18632/oncoscience.609

**Published:** 2024-10-08

**Authors:** Mafalda Costa, Helena Magalhães

**Affiliations:** ^1^Department of Medical Oncology, Hospital Pedro Hispano, Matosinhos, Portugal

**Keywords:** lung cancer, complete response, immune checkpoint inhibitors, brain metastasis, whole-brain radiotherapy

## Abstract

Approximately 20% of lung cancer patients have brain metastasis at diagnosis, which is associated with a worse prognosis and a negative impact on quality of life. The emergence of new systemic treatment options such as immune checkpoint inhibitors (ICI) and targeted therapies changed the prognosis for stage IV lung cancer patients. However, the impact of local and systemic treatment sequencing in patients with stage IV lung cancer and brain metastasis is still unclear. We present the case of a 51-year-old man with stage IV non-small cell lung cancer and brain metastasis at diagnosis who underwent whole brain radiotherapy (WBRT) and achieved intracranial and extracranial complete response after second-line treatment with an ICI. Currently, the patient has an overall survival of 87 months and a progression-free survival of 73 months with an optimal quality of life. We hypothesized that treatment sequencing of WBRT and immunotherapy could explain this unexpected outcome.

## INTRODUCTION

Lung cancer is the leading cause of cancer-related death worldwide, with an estimated 1.8 million deaths (18%) in 2020 [1]. It was responsible for around 2.2 million new cancer cases in 2020, making it the second most common cancer diagnosis after breast cancer [1].

Non-small cell lung cancer (NSCLC) accounts for 80–90% of all lung cancer cases and the vast majority is diagnosed in advanced stages, with an estimated 5-year survival rate of around 18–21% [2]. The recent availability of targeted therapies and immunotherapy with checkpoint inhibitors (ICI) has significantly improved patient prognosis, with a 5-year survival rate ranging from 15% to 50%, depending on the biomarker [3]. Many NSCLC patients experience brain metastasis (30–50%), which significantly impacts their quality of life and prognosis [4, 5]. Treatment options for limited brain metastases in NSCLC patients include stereotactic radiosurgery alone (SRS), surgical resection for selected patients followed by stereotactic radiosurgery, or whole-brain radiotherapy [3]. However, effective and curative treatment of brain metastasis remains challenging for most patients, and more effective therapies are needed.

The role of ICI in brain metastasis is still unclear due to the underrepresentation of these patients in ICI clinical trials. However, some retrospective studies and prospective trials suggest the activity and safety of ICI in patients with brain metastasis. There is limited data regarding the optimal timing and sequencing of different treatment modalities to optimize treatment outcomes in NSCLC patients with brain metastasis at diagnosis [6, 7].

This article reports the case of a patient with stage IV NSCLC with high-volume metastatic disease, including brain metastasis, who achieved a complete response with second-line immunotherapy after whole-brain radiotherapy and first-line chemotherapy.

## CASE PRESENTATION

**Figure 1:**
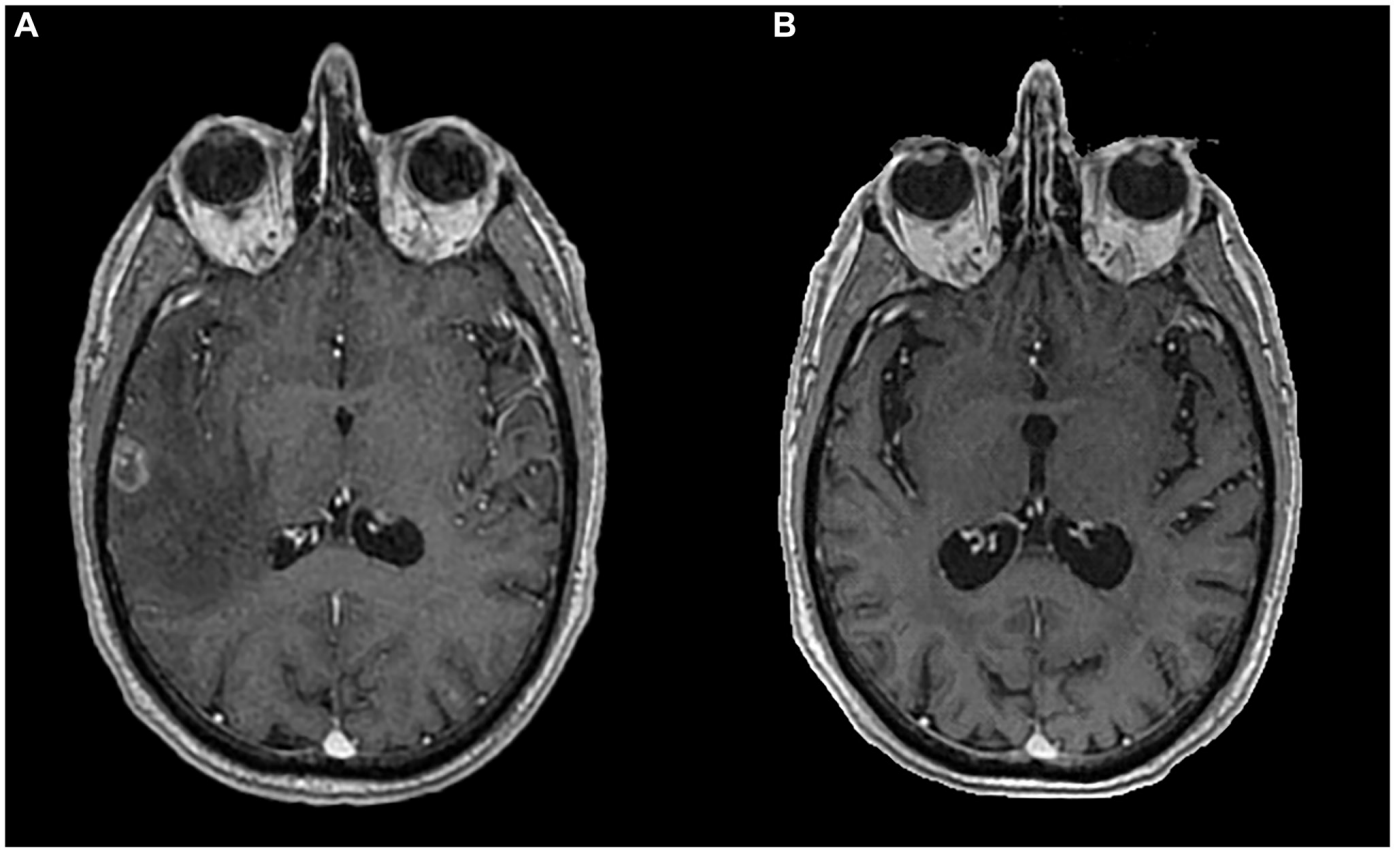
Brain MRI at diagnosis and last Brain MRI performed. The image shows a temporal metastatic lesion at diagnosis (**A**) and last Brain MRI with maintained complete response (**B**), in postcontrast 3D T1-weighted.

**Figure 2:**
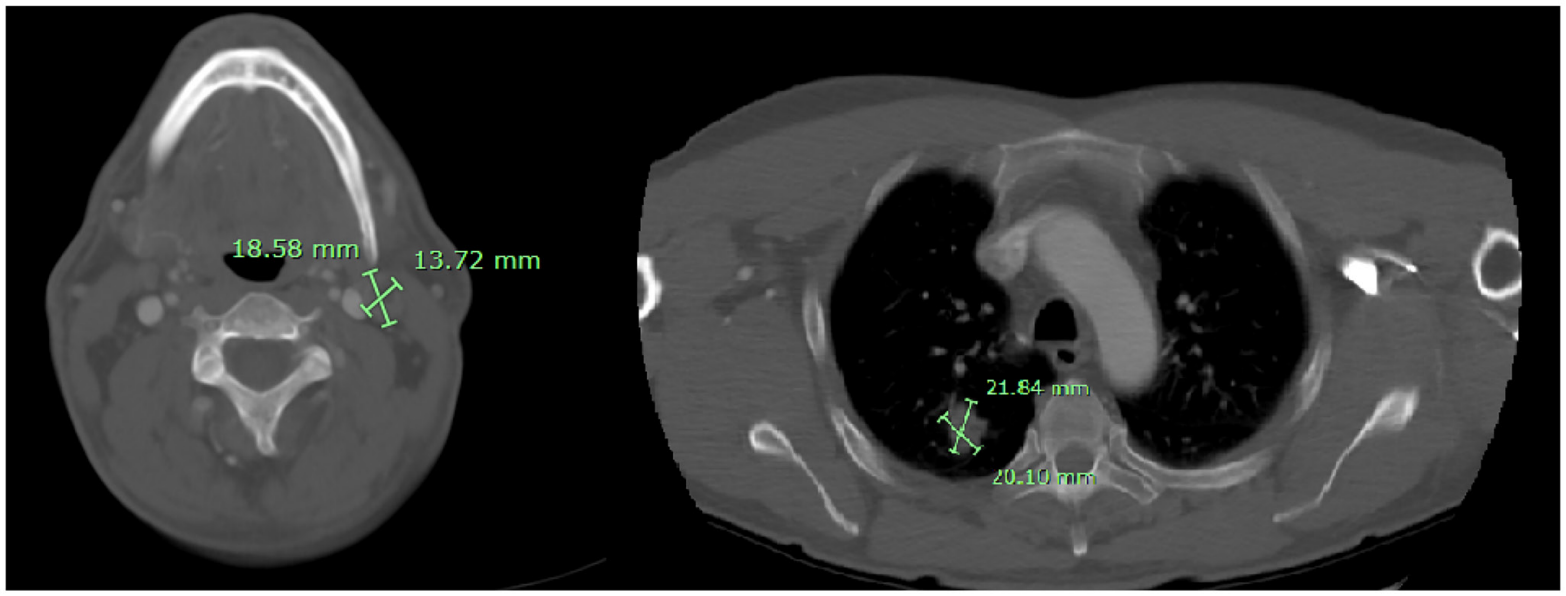
Axial contrast-enhanced CT scan at diagnosis. The image shows a carotid-jugular adenopathy (left), nodular spiculated lesion in the superior right lobe (right), dimensions in green mark.

A man in his early 50s, with smoking habits (35 packs a year) and no other relevant comorbidities, was admitted to the emergency room with a generalized tonic-clonic seizure. A brain magnetic resonance imaging (MRI) revealed 3 lesions in the brain parenchyma. The biggest one dimensioning 16,8 mm located in the temporal lobe, and two smaller lesions in the high frontal and parietal convexity. These lesions were surrounded by vasogenic edema, causing a mass effect and leftward deviation of midline structures (Figure 1A). The patient was started on anti-epileptic treatment and high-dose corticosteroid and was admitted for further clinical investigation. A thoracoabdominal computed tomography (CT) scan showed a necrotic adenopathy of 18 mm in the left carotid-jugular space, as well as a nodular spiculated lesion with 21 mmx20 mm in the upper right lobe (Figure 2). No evidence of lung or pericardial effusion, adrenal or hepatic lesions was found. An examination by an otorhinolaryngologist revealed no evidence of a head and neck tumor.

A transthoracic biopsy of the nodular spiculated lung lesion revealed an adenocarcinoma, with positive immunoexpression for CK7 and TTF1, and negative for CK20. Cytology of the mentioned adenopathy was compatible with metastasis from adenocarcinoma. Bone scintigraphy detected two osteoblastic lesions in D9 and D8 compatible with metastasis. The clinical stage was T1bN3M1c- stage IVB according to the AJCC Cancer Staging 8th edition. Next-generation sequencing did not identify any potential molecular targets, including EGFR mutations or ROS1/ALK rearrangements. Immunohistochemical analysis revealed a PDL1 expression of 70%.

**Figure 3:**
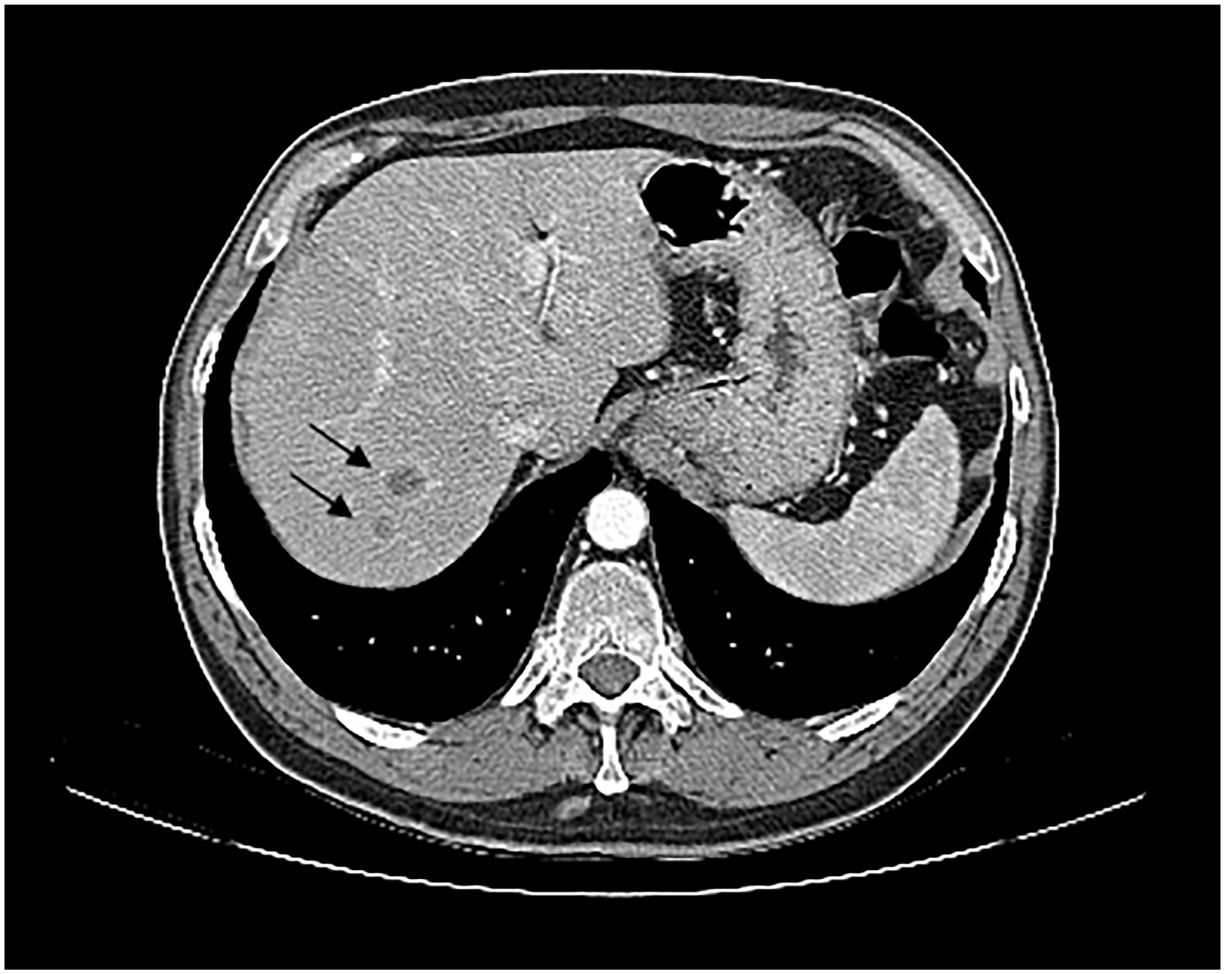
Axial contrast-enhanced CT scan upon progression. Image shows hepatic progression with metastatic lesions not accessible to biopsy (arrow).

Whole brain radiotherapy (WBRT) was performed with 20 Gy in 5 fractions. Due to high corticosteroids on course, the patient didn’t start immunotherapy treatment as planned and instead began chemotherapy with carboplatin 5 AUC and pemetrexed 500 mg/m^2^ every three weeks. The patient showed a partial response to treatment, according to Response Evaluation Criteria In Solid Tumours (RECIST) 1.1 criteria, with disappearance of adenopathy and reduction of the pulmonary nodule to 12x10mm. Subsequently, the patient underwent maintenance pemetrexed completing 4 cycles. At this time, a CT scan showed two new hepatic lesions of 13 and 18 mm in the posterior segment of the right hepatic lobe and several other minor lesions spread in the hepatic parenchyma, highly suggestive of new metastatic lesions that were not possible to biopsy (Figure 3). Brain MRI showed a maintained complete cerebral response (Figure 1B).

The patient then started second-line treatment with pembrolizumab (200 mg intravenously every three weeks). After 3 cycles, a CT scan revealed a partial response, according to immune RECIST criteria, with hepatic lesions measuring 6 and 8 mm and a stable pulmonary lesion. By the 6th cycle, the CT scan showed a complete hepatic response and partial pulmonary response, while the brain MRI confirmed a maintained complete response. Subsequently, there was a progressive slight reduction in the pulmonary primary lesion to a minimum of 10 mm, which was described as a fibrous-cicatricial nodule in follow-up exams and D9 and D8 bone lesions were not active in follow-up bone scintigraphy.

**Figure 4:**
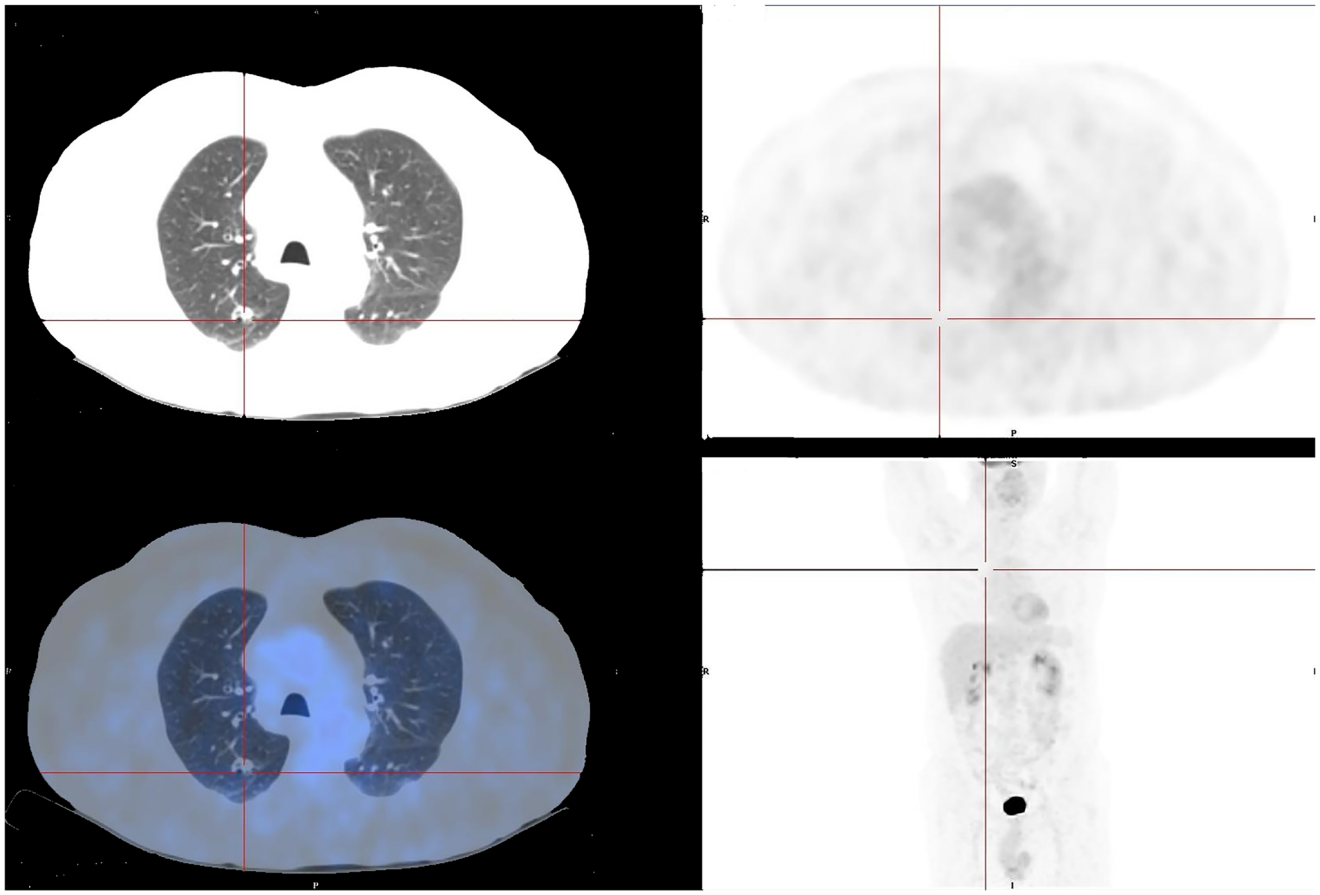
PET/CT-FDG scan one month after pembrolizumab suspension. The image shows a fibrous-cicatricial nodule with no FDG uptake in the superior right lobe.

The patient maintained a cerebral, hepatic, and pulmonary complete response and received a total of 53 cycles of pembrolizumab with good tolerance. The only reported side effect was a grade 1 pruriginous rash, according to Common Terminology Criteria for Adverse Events (CTCAE) classification, managed temporarily with anti-histaminic and topical corticosteroids. A fluorodeoxyglucose-positron emission tomography scan performed one month after treatment suspension did not reveal metabolic active lesions (Figure 4).

At the latest appointment, 26 months after treatment suspension, the patient remains asymptomatic with an excellent quality of life and no evidence of disease. Current overall survival (OS) and progression-free survival (PFS) are 87 months and 73 months, respectively.

## DISCUSSION

In the last decade, immunotherapy agents changed the treatment landscape for Non-Small Cell Lung Cancer (NSCLC). Currently, the standard first-line treatment for stage IV NSCLC without driver mutations involves the use of immunotherapy agents, either alone or in combination with chemotherapy, based on PD-L1 expression [8]. Initially, immune checkpoint inhibitor (ICI) treatment for lung cancer was approved as a second-line option following progression on chemotherapy, according to the results of first published clinical trials with nivolumab and pembrolizumab in monotherapy [9, 10].

The five-year survival follow-up of Keynote 010 (Kn 010) study revealed that patients with PD-L1 TPS ≥50% treated with pembrolizumab instead of docetaxel in the second-line setting had a median overall survival of 16.9 months compared to 8.2 months, and a median progression-free survival of 5.3 months compared to 4.2 months.

In the Kn 010 study, 79 patients (11.4%) completed 35 cycles of pembrolizumab. Among them, 15 patients (19.0%) achieved a complete response, 63 patients (79.7%) achieved a partial response, and one patient (1.3%) had stable disease [10].

After progression of the disease on platinum-based chemotherapy, our patient started immunotherapy as a second-line palliative treatment. The patient with stage IV lung cancer achieved a cerebral, pulmonary, and hepatic complete response sustained for 69 months. This response is maintained 26 months after treatment suspension with pembrolizumab, which, according to available data, is an unexpected outcome.

There are very few case reports in the literature of a complete response to anti-PD-1 agents as first-line treatment of stage IV lung cancer. To our knowledge, there are no case reports of a complete response in the second-line setting, and specifically, no similar case with a sustained cerebral complete response for such a long duration. Some particularities of this case and its treatment sequence could explain this excellent response and will be further explored.

Only a small number of patients experience long-lasting responses to immunotherapy. Ongoing efforts are being made to identify predictive biomarkers of response both host and tumor-related. This will help identify patients who will benefit the most from treatment and avoid potential side effects in those who may not respond to it. Currently, the only available biomarker predictor of response to single-agent anti-PD-1/PD-L1 treatment is the expression of PDL-1 in the tumor and its microenvironment. However, PD-L1 expression alone is not a strong predictor of treatment response. Other biomarkers are being studied as predictors of response to ICI [11]. In this case, the patient had a high expression of PDL-1 (70% by tumor proportion score) which is known from phase III clinical trials to predict a better response to anti-PD-1 agents. However, other clinical, pathological, and immune-related factors most likely have played a significant role in such a long-lasting complete response (69 months).

In addition, our patient had brain metastasis and was submitted to WBRT 8 months before starting second-line treatment with pembrolizumab. It is understood on a biological level that there is a time and spatial-dependent synergic effect between immunotherapy (IT) and radiotherapy (RT).

On one hand, RT can modulate tumor behavior by damaging cancer cells and exposing more tumor antigens to dendritic cells, which in turn leads to increased recruitment of CD4+ and CD8+ T cells. On the other hand, RT also activates several important inflammatory pathways.

Combining RT with IT results in a stronger immune response against tumor cells and enhances the efficacy of IT [12]. Further research is needed to determine the best way to combine and sequence RT and IT to maximize the synergistic effects of these two treatments.

There is limited data on the impact of IT on brain metastasis, specifically regarding the permeability of pembrolizumab at the blood-brain barrier. The brain metastases microenvironment has unique characteristics in terms of immune surveillance, with a higher number of tumor-infiltrating lymphocytes, which sets it apart from other metastatic sites. Some early clinical trials and case reports suggest potential activity and response to IT in brain metastasis, especially when combined with other treatments such as WBRT, SRS, or surgical resection [13]. However, patients with advanced NSCLC and active brain metastases are typically excluded from clinical trials [14]. In a retrospective pooled analysis of Keynote 001, 010, 024, and 042, pembrolizumab monotherapy improved the outcome of patients with NSCLC stage IV, regardless of the presence of treated, stable brain metastases at baseline, with a greater benefit seen in patients with PD-L1 TPS >50% [15]. Several studies tried to address the best treatment sequencing and cerebral RT modality in patients with NSCLC and brain metastasis, showing promising results in terms of safety and efficacy [16]. However, further studies are required to accurately predict which patients with NSCLC and brain metastasis will respond to IT.

We have limited knowledge about which factors influence response to IT in NSCLC and the potential interactions between different treatments, as well as the impact of treatment sequencing on treatment response. In this case, it’s uncertain whether WBRT followed by pembrolizumab enhanced a sustained complete response in the brain and led to a similar response in other metastatic sites, which could be a possible explanation for this outcome. Other questions raised by this clinical case, were when to discontinue immunotherapy after achieving a complete response and whether pembrolizumab should be reintroduced after disease progression. In the KN 010 trial, pembrolizumab was continued for 24 months or until disease progression or severe toxicity [10]. Clinicians have wondered about the optimal timing for treatment suspension in patients with complete responses, as there is no guidance from clinical trials.

A recent retrospective cohort study addressed the question if patients with NSCLC who have a long-term response to IT should stop treatment at 2 years or continue indefinitely. This study found no statistically significant difference in overall survival between patients who continued IT treatment indefinitely and those who stopped treatment after 2 years (2-year OS of 81 vs. 79%). This suggests that it may be safe to stop treatment at 2 years for patients who obtained a sustained response. However, the study noted that only about 1 in 5 patients stopped IT at 2 years without disease progression [17].

In this case, the patient stopped treatment after 53 cycles of pembrolizumab and continued treatment for 41 months after achieving a complete response. We decided to continue treatment due to the lack of data supporting our decision to stop at that time and because of the patient’s preferences.

Regarding the reintroduction of pembrolizumab upon progression, the Kn 010 study showed that 52.4% of patients who received a second course of pembrolizumab responded to treatment. However, only a small number of patients (*n* = 21) were submitted to rechallenge [10]. A recent meta-analysis of ICI rechallenge in NSCLC patients showed that those who achieved long-term remission for more than 2 years after ICI treatment had a higher objective response rate (40–60%) upon ICI rechallenge. This suggests that ICI rechallenge could be an option for our patient, but more data is needed to identify predictive factors for response to ICI rechallenge [18].

This unique case highlights the potential for achieving a complete response with ICI monotherapy as second-line treatment of stage IV NSCLC with brain metastasis at diagnosis. The treatment combination and sequencing may have contributed to an improved treatment efficacy, leading to a durable complete response, long-term survival (87 months), and maintained quality of life.
